# Cross-linguistic syntactic priming as rational expectation for syntactic repetition in the bilingual environment

**DOI:** 10.1371/journal.pone.0307504

**Published:** 2024-07-19

**Authors:** Kexin Xu, Tao Zeng

**Affiliations:** 1 College of Foreign Languages, Hunan University, Changsha, China; 2 Hunan Provincial Research Center for Language and Cognition, Changsha, China; Potsdam University, GERMANY

## Abstract

Recent research suggests that syntactic priming in language comprehension—the facilitated processing of repeated syntactic structures—arises from the expectation for syntactic repetition due to rational adaptation to the linguistic environment. To further evaluate the generalizability of this expectation adaptation account in cross-linguistic syntactic priming and explore the influence of second language (L2) proficiency, we conducted a self-paced reading study with Chinese L2 learners of English by utilizing the sentential complement-direct object (SC-DO) ambiguity. The results showed that participants exposed to clusters of SC structures subsequently processed repetitions of this structure more rapidly (i.e., larger priming effects) than those exposed to the same number of SC structures but spaced in time, despite the prime and target being in two different languages (Chinese and English). Furthermore, this difference in priming strength was more pronounced for participants with higher L2 (English) proficiency. These findings demonstrate that cross-linguistic syntactic priming is consistent with the expectation for syntactic repetition that rationally adapts to syntactic clustering properties in surrounding bilingual environments, and such adaptation is enhanced as L2 proficiency increases. Taken together, our study extends the expectation adaptation account to cross-linguistic syntactic priming and integrates the role of L2 proficiency, which can shed new light on the mechanisms underlying syntactic priming, bilingual shared syntactic representations and expectation-based sentence processing.

## Introduction

Repetition of syntactic structures facilitates language comprehension. That is, a sentence is processed faster if it repeats the syntactic structure from a previously encountered sentence. This phenomenon is known as “syntactic priming” during comprehension because it does not depend on processing strategies [1] or repetitions of non-syntactic (e.g., lexical, semantic, etc) items [2,3] (although these repetitions can usually strengthen priming effects [4]), which suggests the existence of abstract syntactic representations as meaningful processing units. Syntactic priming has been demonstrated to occur in a wide range of syntactic structures and languages by using a variety of experimental paradigms and tasks [5–10] (for a recent review, see Tooley [11]). The universality of this phenomenon prompts the inquiry into its underlying mechanisms: why do comprehenders process repeated structures more rapidly than novel constructions?

One recent influential account proposed by Myslín and Levy [12] posits that syntactic priming is a result of the expectation for syntactic repetition due to rational adaptation to the linguistic environment (see the “Literature Review” section for more details about this account). This expectation adaptation account creatively integrates syntactic priming into the predictive, dynamic system of expectation-based language comprehension. More importantly, it contributes to the mechanistic understanding of syntactic priming by pointing out the role of linguistic environment, an aspect that many previous accounts fail to adequately address. However, despite its significant contributions and merits, this account faces limitations in generalization since it has garnered empirical support only from within-language syntactic priming [12,13]. In fact, syntactic priming has been widely observed not only within the same language but also across two different languages [14–17], with its effects notably affected by second language (L2) proficiency [18–21]. These findings raise further intriguing and yet unexplored questions about whether the mechanism of expectation adaptation also holds for cross-linguistic syntactic priming, and how L2 proficiency may modulate this mechanism.

The present study aims to address these questions by employing the self-paced reading paradigm of Myslín and Levy [12] and extending it to a bilingual context with Chinese L2 learners of English. Our findings would be informative for mechanisms behind cross-linguistic syntactic priming, bilingual shared syntactic representations, and expectation-based sentence processing. Before delving into the experiment, we first review previous studies on syntactic priming, placing particular emphasis on its mechanistic accounts and cross-linguistic characteristics.

## Literature review

### Syntactic priming as residual activation or implicit learning

Most previous mechanistic accounts of syntactic priming fall into two broad classes: residual activation and implicit learning. Simply put, the residual activation account holds that accessing a syntactic structure increases the mental activation level of its structural nodes that are connected to verbs, resulting in temporarily facilitated processing for subsequent tokens of this structure [22]. This activation-based account is supported by the short-term priming that rapidly decays as short as one intervening unrelated sentence between the prime and target [23,24], and also by the lexical boost effect (i.e, priming strength is enhanced when the prime and target share the same verb) that indicates the further activation of the connection between structural nodes and verbs [25,26]. In contrast, the implicit learning account argues that processing a syntactic structure brings about the unconscious learning of its structural knowledge, leading to relatively prolonged facilitation for subsequent processing of this structure [27]. This learning-based account is corroborated by the long-term priming that can persist across a few intervening unrelated sentences [28], an entire experimental phase [29], and even several days [30]. Despite their different perspectives concerning the mechanisms of syntactic priming, these two accounts are not mutually exclusive but rather complementary: the short-term and lexically dependent priming is caused by residual activation, whereas the long-term and lexically independent priming is driven by implicit learning (i.e., the dual mechanism account) [31,32].

### Syntactic priming as expectation adaptation

Building on the implicit learning account at the computational level of analysis, the expectation adaptation account seeks to explain the adaptive nature of syntactic priming. The term “expectation” can be generally defined as the subjective estimated probability of observing particular linguistic properties (e.g., sounds, words, syntactic structures, etc) in forthcoming input; it is often used interchangeably with the term “prediction” or “anticipation” (for discussion on this matter, see Kuperberg and Jaeger [33]). Starting with the premise that comprehenders are able to generate expectations for upcoming syntactic structures based on prior language experience (the surprisal theory) [34,35], and given the goal of achieving accurate and efficient online sentence processing in real-time, a rational behavior for comprehenders is to rapidly adapt their expectations to converge towards or even on syntactic distributions in surrounding linguistic environments [36].

Syntactic priming may be a consequence of the rational expectation adaptation, as preliminarily evidenced by two well-documented effects: the inverse frequency effect and the cumulative effect. The inverse frequency effect refers to the phenomenon that less frequent structures are associated with stronger priming effects than their more frequent alternatives (e.g., English speakers were primed more by the less frequent propositional object (1a) than the more frequent double object (1b)) [37–39]. This effect implies that encountering the less frequent structure, which is less expected on the basis of prior language experience, prompts comprehenders to more dramatically raise their expectations for this structure, thus eliciting stronger priming effects. The cumulative effect further extends this trial-to-trial adaptation to a continuous level by manifesting that priming effects can accumulate over exposures to critical structures (e.g., repeated exposures to reduced relative primes (2a) led to cumulative reductions in processing difficulty of those structure targets (2b)) [40–42]. This effect indicates that the more frequently a structure is encountered in recent input, the more strongly comprehenders come to expect that structure, thereby resulting in the accumulation of priming effects. Taken together, these findings suggest that the variability in priming strength may reflect the adjustment of expectation level.

1a. John gave a book to his daughter.
1b. John gave his daughter a book.
2a. The soldiers warned about the dangers conducted the midnight raid.
2b. The workers warned about the wages decided to file complaints.

The connection between expectation and syntactic priming has led Myslín and Levy [12] to propose the expectation adaptation account. According to this account, syntactic priming results from the expectation for syntactic repetition due to rational adaptation to the linguistic environment. To illustrate, natural language is a clustering environment where tokens of the same syntactic structures frequently occur in closer succession than predicted by chance, as evidenced by many corpus studies [43–45] (for a review, see Gries and Kootstra [46]). Given such syntactic clustering properties in natural language, it is rational for comprehenders to expect another instance of a structure closely following its first occurrence. Where possible, then, it is adaptive for comprehenders to keep track of syntactic clustering properties in current linguistic environments, coming to more strongly expect the repetitive occurrence of a structure in environments where it clusters than in environments where it does not. Accordingly, syntactic priming is a result of the rational expectation for syntactic repetition, which is initially motivated by syntactic clustering properties in natural language and then is modulated by such properties in current linguistic environments.

Myslín and Levy [12] tested this expectation-based account by making use of the sentential complement-direct object (SC-DO) ambiguity in English. In this ambiguity, the post-verbal noun phrase “the solution” can either be the subject of a sentential complement (3a) or the direct object of the verb (3b). They first presented a corpus study showing that occurrences of SC structures indeed clustered in natural language, which motivated an expectation for repetition of this structure. They then carried out two between-group self-paced reading experiments, which demonstrated the rational adaptation of such expectation. In these experiments, two groups of English native speakers read two-sentence vignettes in which SCs occurred either successively (the clustered group) or non-adjacently (the anti-clustered group) in a training phase, with the total number of SCs being the same for both groups. Afterwards, all participants read the same repetitions of SCs in a test phase. It was found that participants in the clustered training group were more facilitated to process repeated test SCs than those in the anti-clustered training group. This difference in priming strength suggests that participants rationally adapt their expectation for repetition of SC structure to match its environmental clustering properties. In addition, such difference still held when the two-sentence vignettes were embedded in longer contexts ranging from three to five sentences in length, ruling out an alternative possibility that participants simply adapt to the paired nature of stimulus rather than the linguistic property of environments.

Similar results were obtained by Han [13] who replicated the self-paced reading paradigm with Chinese native speakers. As the SC-DO ambiguity can also be found in Chinese (e.g., the post-verbal noun phrase “解决方案” (the solution) can be treated either as the subject of a sentential complement (4a) or as the direct object of a verb (4b)), Han [13] translated the English materials of Myslín and Levy [12] into Chinese and maintained the same design to test the rational expectation adaptation in Chinese syntactic priming. Han [13] found that participants trained in environments where SCs clustered similarly exhibited larger priming effects for this structure in the subsequent test compared to those trained in environments where SCs did not cluster, which again corroborated the expectation adaptation account.

3a. Her friend whispered the solution was to dispose of the evidence. (SC)
3b. Her friend whispered the solution very quietly in her ear. (DO)
4a. 他说解决方案是复查案件 (SC)

‘He said the solution was to reexamine the case.’

4b. 他说解决方案时非常小心 (DO)

‘He said the solution very carefully.’

While the above between-group difference in priming strength is in line with the expectation adaptation account, it is not predicted by previous accounts of syntactic priming. According to these accounts, the same total number of SCs experienced by both groups in the training phase should achieve the comparable activation level of structural nodes (the residual activation account) or learning outcome of structural knowledge (the implicit learning account), leading to equal priming strength for this structure in the test phase. Therefore, the expectation adaptation account serves as a crucial complement to existing mechanistic accounts by incorporating the modulation of environmental clustering properties on priming strength. Additionally, this account situates syntactic priming within the broader system of expectation-based language comprehension by pinpointing its adaptive and context-sensitive nature. Nevertheless, while this account has been empirically verified in within-language syntactic priming, its generalizability to cross-linguistic syntactic priming remains unexplored.

### Syntactic priming as a cross-linguistic effect

Syntactic priming has long been established as a cross-linguistic effect that can take place between two different languages during comprehension, which is taken as evidence for shared syntactic representations in the bilingual mind. For example, in a self-paced reading study with Chinese L2 learners of English, Hsieh [14] discovered that English passive relative targets (5c) were read faster when following Chinese passive relative primes (5b) than active main primes (5a). In addition, cross-linguistic priming effect was recorded in other language pairs by using other measurements as well, such as the stronger tendency to choose pictures with German object relative interpretations after reading English object relative sentences compared to subject relative sentences in a sentence-picture matching task [15], the higher proportion of looks to target scenes that depict English object-question events after exposures to German or Japanese object-question primes than subject-question primes in a visual world paradigm [16], and the decrease in brain activity following the repetition of passive structures from German to English in an fMRI (functional magnetic resonance imaging) experiment [17].

5a. 那个认真负责的警察昨天逮捕了小偷

‘The earnest, responsible policeman arrested the thief yesterday.’

5b. 那个被警察逮到的小偷感到很羞愧

‘The thief arrested by the policeman felt ashamed.’

5c. The designer praised by the client was very creative.

Does the expectation adaptation account also extend to cross-linguistic syntactic priming? Despite the lack of direct investigations, there are several indirect evidence supporting this extension. To start with, many corpus studies have found that syntactic distributions in spontaneous bilingual dialogue are also subject to a certain degree of clustering, in that the repetition of a structure in one language is strongly predicted by its initial occurrence in another language (especially in the form of code-switching) [47–50]. These syntactic clustering properties in natural bilingual speech provide the initial motivation for generating cross-linguistic expectations for syntactic repetition. Additionally, numerous priming studies have shown that less frequent structures in L1 elicit stronger priming effects in L2 than their more frequent alternates [51–53], and repeated exposures to a structure in L1 (or L2) lead to accumulated priming effects for that structure in L2 (or L1) [54–56]. Such bilingual versions of the inverse frequency effect and the cumulative effect demonstrate that current environmental distributions in one language can affect priming strength in another language, which suggests the rational adaptation of expectations across languages. Given these findings, a primary goal of this study is provide a direct test on the validity of the expectation adaptation account in cross-linguistic syntactic priming.

Furthermore, cross-linguistic syntactic priming has been reported to be notably influenced by L2 proficiency. For instance, in two priming experiments with Dutch L2 learners of English who possessed varying L2 proficiency, Bernolet et al. [18] observed stronger cross-linguistic priming effects for high- than low-proficiency L2 learners. In another study with Dutch L2 learners of English, Hartsuiker and Bernolet [19] found that cross-linguistic priming effects were stronger with more proficient L2 learners as well. More recently, the similar proficiency pattern was also identified by Hwang et al. [20] with Korean L2 learners of English and by Favier et al. [21] with English L2 learners of Irish. This positive relation between cross-linguistic syntactic priming and L2 proficiency may be ascribed to the stronger ability of more proficient L2 learners to generate cross-linguistic expectation for syntactic repetition. Following this logic, it is reasonable to further infer that the ability of L2 learners to adapt such cross-linguistic expectation may also be enhanced with the increase of L2 proficiency, as more balanced bilinguals may be more capable of leveraging the knowledge of clustering properties in current linguistic environments. In light of this, this study adds L2 proficiency as an influencing factor.

### The present study

In the present study, we aim to explore (1) whether the mechanism of expectation adaptation extends to cross-linguistic syntactic priming and (2) how this mechanism may be modulated by L2 proficiency. To this end, we employ the self-paced paradigm of Myslín and Levy [12] that utilizes the sentential complement-direct object (SC-DO) ambiguity, and modify it to a bilingual context by presenting the prime and the target in two different languages: Chinese and English. The rationale for choosing these two languages is two fold: First, the SC-DO ambiguity similarly exists in both English and Chinese (see example sentences 3–4), making them ideal for comparing effects of this structural ambiguity across languages. Second, given that the expectation adaptation account has been verified in syntactic priming within English [12] and within Chinese [13], our exploration into this account’s generalizability to syntactic priming between English and Chinese is not constrained by concerns of its within-language validity.

Specifically, in our experiment, two groups of Chinese L2 learners of English read the same total number of SCs, but experience different clustering properties (SCs always cluster for one group, but never cluster for another group) in a training phase. Afterwards, all participants read clusters of two SCs in a test phase. If the expectation adaptation account extends to cross-linguistic syntactic priming, then we expect the group with clustered training experience to be more facilitated to process the second sentence in test SC clusters (i.e., exhibited larger priming effects) than the group with anti-clustered training experience, despite the prime and target being in two different languages (Chinese prime and English target or English prime and Chinese target). Moreover, if L2 proficiency exerts a positive influence on the expectation adaptation in cross-linguistic syntactic priming, then we expect the between-group difference in priming strength to be enlarged as L2 proficiency increases.

## Methodology

### Participants

A total of 144 college students (100 female, 44 male) with an average age of 20.57 years (SD = 1.75; range = 18–26) from Hunan University in China took part in the experiment. They were all native Chinese speakers who started learning English as a second language after the age of six, with no experience of living or studying abroad. All of them reported no history of reading or cognitive disabilities, and had normal or corrected-to-normal vision. Their L2 proficiency was measured by using the Oxford Quick Placement Test (OQPT), a standardized English test that consists of 60 questions (one point per question). We excluded four participants from the analysis because of their OQPT scores being too low (less than 18, a threshold classified as the elementary level). The mean OQPT score of all remaining participants was 41.36 (SD = 8.56; range = 22–58), which fell into the upper intermediate level.

Participants were assigned to one of the two priming directions (half of them to the Chinese-to-English and the other half to the English-to-Chinese), and within each direction they were further divided into either the clustered or anti-clustered training conditions, based on the criterion of maintaining comparable L2 experience across all conditions. Participants’ L2 information was summarized in Table 1. ANOVA tests showed no significant differences in OQPT scores (*F*(3, 136) = 0.025, *p* = 0.995), age of acquisition (*F*(3, 136) = 1.982, *p* = 0.120), or length of exposure (*F*(3, 136) = 0.689, *p* = 0.560) across the four groups of participants.

**Table 1:** Participant L2 information by priming direction and training condition.

	Chinese-to-English		English-to-Chinese	
	Clustered	Anti-clustered	Clustered	Anti-clustered
Number	35	35	35	35
OQPT score	41.37 (8.84)	41.66 (8.67)	41.09 (8.61)	41.34 (8.12)
Age of acquisition (years)	8.86 (0.76)	8.80 (0.89)	8.46 (0.97)	8.91 (0.77)
Length of exposure (years)	11.57 (1.71)	11.63 (1.64)	11.94 (1.41)	11.40 (1.59)

*Note*. Means are shown with standard deviations in parentheses.

Approval of this research was granted by the Human Research Ethics Committee of Hunan University. Written informed consent was obtained from all participants in compliance with the experiment protocols.

### Materials

Stimuli were two-sentence vignettes, where each sentence contained either a sentential complement (SC), double object (DO), or other unrelated structure (OTH). Order of sentences within vignettes was constant throughout the whole experiment. Within each vignette, the two sentences were presented in two different languages: in the Chinese-to-English priming direction, the first sentence (prime) was in Chinese and the second sentence (target) in English, whereas the language order was reversed in the English-to-Chinese priming direction (i.e., English prime and Chinese target). The sentences between two priming directions were translation equivalents to ensure the semantic consistency. English sentences were adapted from Myslín and Levy [12] and translated into Chinese by a professional translator. The syntactic naturalness and comprehensibility of all Chinese sentences were rated by 10 Chinese native speakers who did not participate in the experiment on a 4-point scale. Any Chinese sentence scoring below 2.5 in either syntactic naturalness or comprehensibility, as well as its original English version, was excluded. In total, 92 vignettes (22 SC.SC, 8 SC.DO, 14 SC.OTH, 14 OTH.SC and 34 OTH.OTH) were used as experimental materials in each priming direction. Sample stimulus in both English and Chinese are provided in Table 2. Regions of interest for critical structures are recorded in Table 3.

**Table 2:** Sample stimulus in both English and Chinese.

Structures	Sentences
(1) SC.SC vignette	
SC	Kelly proposed the solution should be more cost-effective 凯莉提出这个方案应该更加划算
SC	The boss confirmed the expense was greater than necessary 老板确认这笔费用是超出所需的
(2) SC.DO vignette	
SC	Monroe checked his wallet was still in his pocket 门罗检查他的钱包是还在口袋里的
DO	He discovered a credit card when opened the wallet 他发现一张银行卡当打开钱包时
(3) OTH.SC vignette	
OTH	The biscuits Natalie baked turned out extremely dry and hard 娜塔莉烤的饼干又干又硬
SC	She had forgotten the procedure had called for adding water 她已经忘记了这个流程需要加水
(4) SC.OTH vignette	
SC	Mary had expected the light could be explained by the scientists 玛丽曾期待这种光能被科学家解释
OTH	Ever since childhood she had always been a very logical person 她从小就是一个很有逻辑的人
(5) OTH.OTH vignette	
OTH	Henry sat in the room watching the kids play in the rain 亨利坐在房间里看着孩子们在雨中玩耍
OTH	His mom never let him go outside in bad weather 他的妈妈从不让他在恶劣的天气外出

**Table 3:** Regions of interest for critical structures.

	INTRO	VERB	AMBIC NP	DISAMBIC	SPILL	CONCLUSION
SC	Monroe	checked	his wallet	was	still	in the pocket
	门罗	检查	他的钱包	是	还在	口袋里的
DO	He	discovered	a credit card	when	opened	the wallet
	他	发现	一张信用卡	当	打开	钱包时

*Note*. Regions of interest included introduction, main verb, ambiguous noun phrase, disambiguation, spillover, and conclusion. The critical regions were the disambiguation and spillover: disambiguation was the first word following the ambiguous noun phrase, which revealed whether the structure was an SC or DO; spillover was the first word or phase following this region.

**Fig 1:**
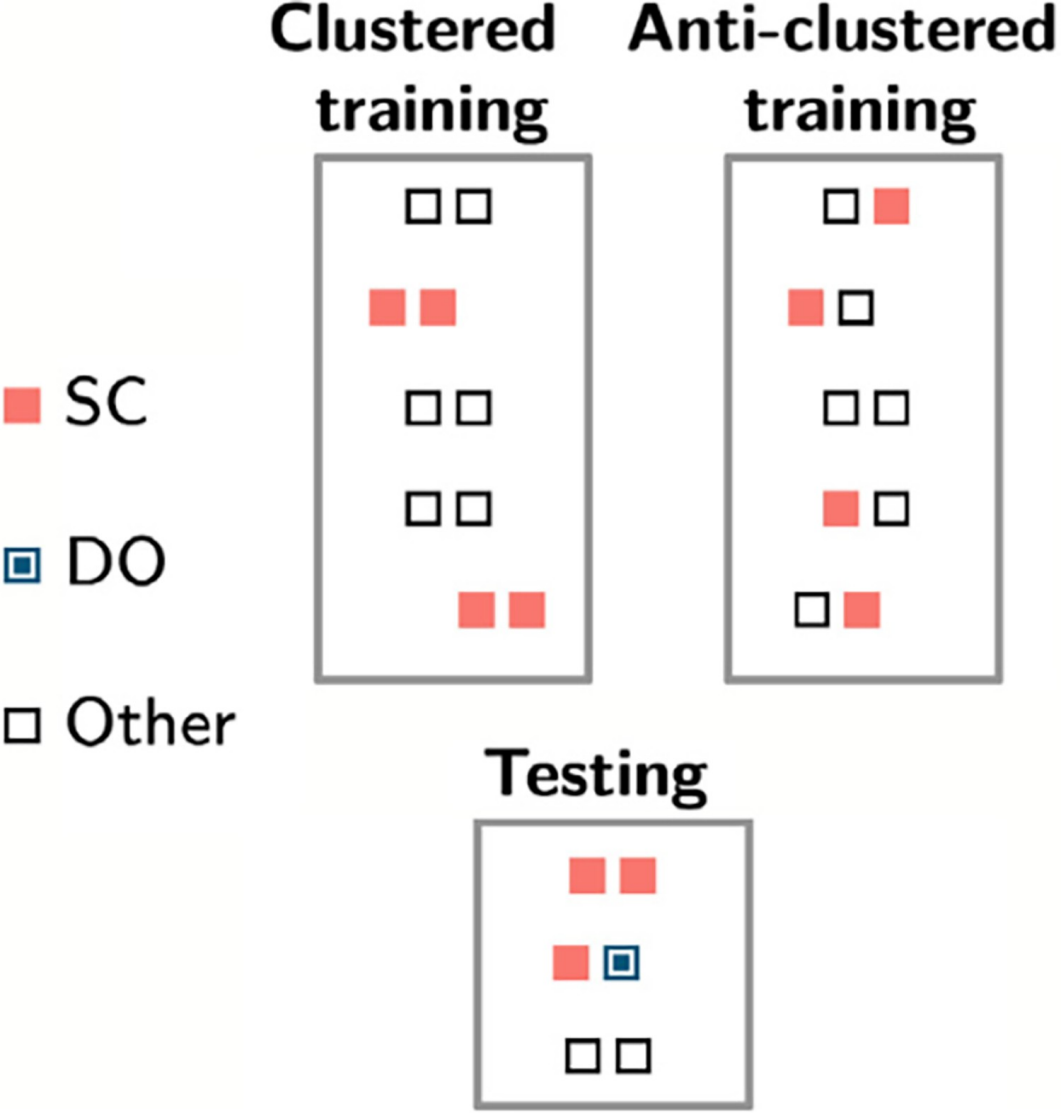
The layout of stimulus in the training and test phase.

The experiment included a training phase and a test phase (see Fig 1). In the training phase, participants were divided into two different training conditions. In the clustered training condition, participants read 14 SC.SC vignettes and 21 OTH.OTH vignettes in which SCs always clustered. Conversely, in the anti-clustered training condition, participants read 14 SC.OTH vignettes, 14 OTH.SC vignettes, as well as 7 OTH.OTH vignettes in which SCs never clustered. Despite the different clustering properties of SCs, the total number of SCs was identical in both conditions. Then, in the test phase, all participants were required to read clusters of SCs which contained 8 SC.SC vignettes, 8 SC.DO vignettes, and 6 OTH.OTH vignettes. In both phases, filler sentences took the vignette type of OTH.OTH, and the ratio of SCs to OTHs was 2:3. Each vignette was followed by a true/false comprehension question to make sure that participants were engaged with materials, and the number of true and false answers was balanced across vignettes types. In addition, each SC or DO sentence contained an unique critical verb, and the mean SC bias of the verbs (i.e., the proportion of verbs that led to an SC structure) was balanced for both English (0.38 in Brown Corpus) and Chinese sentences (0.36 in BLCU Corpus Center). Further, the average word length was equivalent across sentence types (SC, DO, OTH) in both languages.

### Procedure

Stimuli were presented to participants using a word-by-word self-paced reading in which participants pressed the spacebar to reveal each successive word. Reading times were thus defined as the duration between a word’s appearance and the next spacebar press. Each sentence of a given vignette was presented as a new line of text, with the first sentence disappearing when the second sentence appeared. The order of vignettes within the training and testing phase was randomized for each participant. After each vignette, a statement with relevant content was shown, and participants were required to judge whether this statement was true or false. Prior to the formal experiment, participants were given detailed instructions and were provided with 5 practice vignettes (which were not included in the formal experiment) to familiarize themselves with the experimental procedure. An average experimental session lasted about 45 minutes.

Compared with Myslín and Levy [12], we made the following methodological refinements in our experiment. First, we did not retain the first sentence of a vignette visible after the second sentence appeared, as the lingering first sentence might affect participants’ reading of the subsequent sentence. Second, we presented all stimulus in word-by-word reading, instead of presenting 1/4 of stimulus word-by-word and the remaining 3/4 of them sentence-by-sentence, in order to avoid potential disruptions to participants’ reading pace from abrupt presentation changes. Third, for each vignette we added a comprehension question to make sure that participants were engaged with materials. Fourth, we excluded the Conclusion part from critical regions because the well-documented wrap-up effect (i.e., the increased reading time typically observed at the ends of sentences) [57] might confound the adaptation effect being investigated.

## Results

### Data analysis

Following Myslín and Levy’s [12] data analysis approach, participants’ reading times (RTs) at critical regions (disambiguation and spillover) on the continuation (the second sentence) of critical vignettes (SC.SC, SC.DO) in the test phase were analyzed. Data of six participants were deleted due to their low accurate rate of judgement on comprehension questions (less than 60%), which accounted for 4.29% of the data. All raw RTs that were abnormally low (under 100 ms) or abnormally high (over 5000 ms) were excluded, which represented 0.37% of the data. Outliers more than 3 standard deviations from the overall mean for each critical region in each priming direction were removed, which resulted in an additional 1.54% data loss. Remaining RTs were residualized to correct for the influence of word length and individual reading speeds: for each participant, a linear model was fitted between word length (in characters) and reading time, and residual distances were computed between observed RTs and those predicted by the linear model. The residual RTs served as the dependent variable for all subsequent analyses reported below.

**Fig 2:**
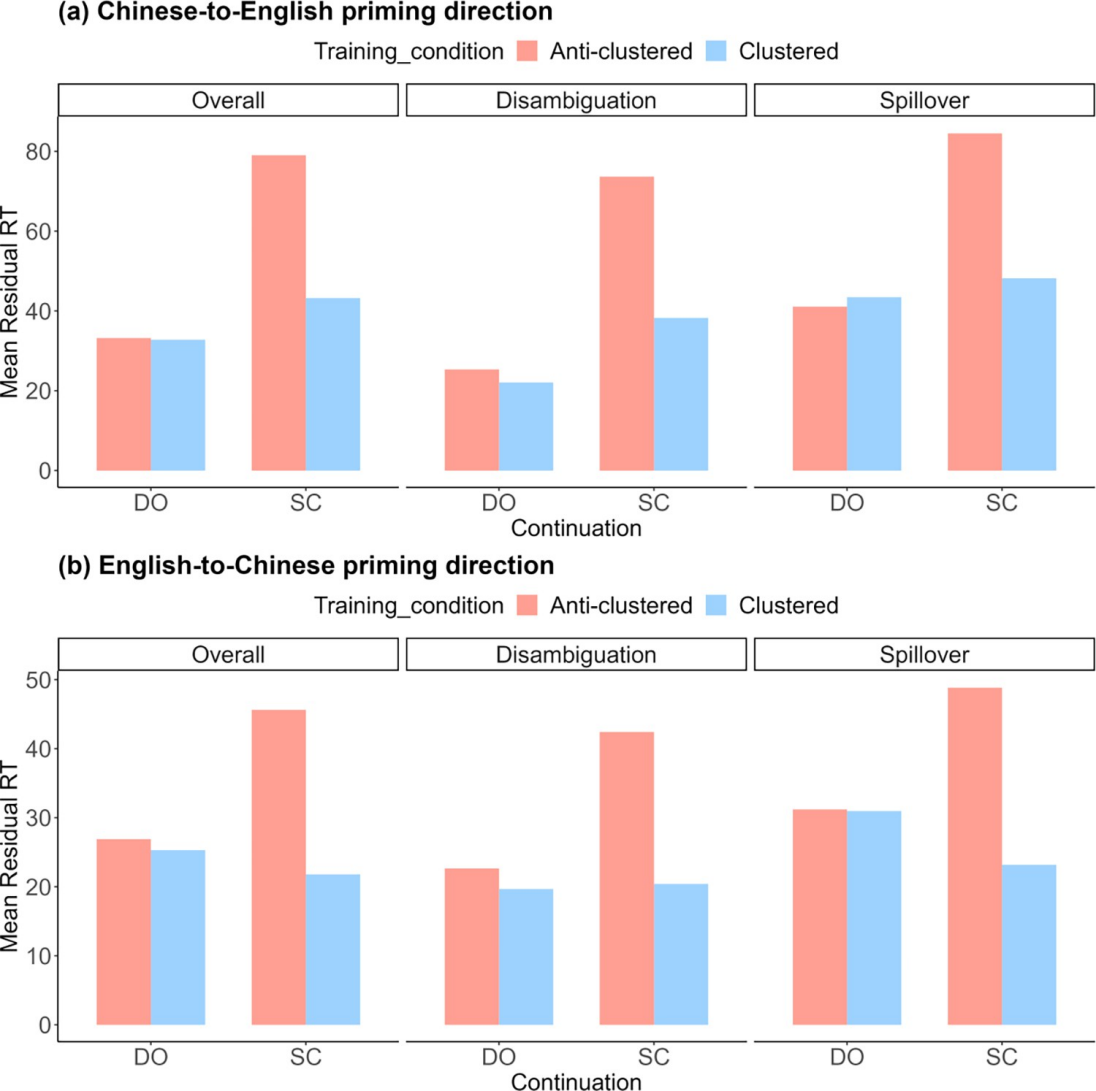
Mean residual RTs at critical regions on continuation of critical test vignettes for both Chinese-to-English (a) and English-to-Chinese (b) priming directions. These figures present the 2*2 pattern of residual RTs as a function of (1) clustered vs. anti-clustered training condition and (ii) SC vs. DO continuation, first averaged over both disambiguation and spillover regions and then broken out separately for each region.

For each priming direction, a linear mixed-effects model was fitted using the lm4 package in R, version 4.3.2 [58]. Fixed effects were training condition (clustered or anti-clustered) and continuation (SC or DO). Random effects were kept as maximal as justified by the experimental design [59], which included subject- and item-specific intercepts, by-subject random slopes for continuation, and by-item random slopes for training condition, as well as their interaction. We used sum coding for binary fixed effect predictors and centered OQPT score as one continuous predictor. We reported coefficient estimates *β* and corresponding *t*-values (|*t|* > 2 indicated a significant effect at approximately *p* < 0.05 [60]). Mean residual RTs at critical regions on continuation in critical test vignettes for two priming directions are shown in Fig 2. Results of linear mixed-effects models are presented in Table 4.

**Table 4:** Results from linear mixed-effects models in two priming directions.

	Chinese-to-English		English-to-Chinese	
Predictor	Coef. *β*	*t*	Coef. *β*	*t*
Intercept	47.06	4.59	20.37	6.81
L2 proficiency	-16.10	-2.67	-8.01	-2.18
Continuation [DO = -0.5, SC = 0.5]	14.06	2.41	8.56	2.33
Training [anti-clustered = -0.5, clustered = 0.5]	1.50	0.29	2.08	0.44
Continuation: Training	-13.84	-2.34	-7.92	-2.07
Continuation: Training: L2 proficiency	-11.10	-2.15	-7.96	-2.09

### RTs by training condition and priming direction

We observed a main effect of continuation, such that RTs for SC continuations were higher than DO continuations in both Chinese-to-English (*β =* 14.06; *t* = 2.41) and English-to-Chinese (*β* = 8.56; *t* = 2.33) priming directions. This observation accorded with the well-known garden-path effect (also termed the ambiguous effect): the temporarily ambiguous structure like SC usually requires a revision of structural analysis (the post-verbal noun tend to be first interpreted as a object of a main clause, and then revised into a subject of an embedded clause, as in example 3a), and thus is processed more slowly than its unambiguous alternation like DO [61]. More crucially, we observed an interaction of training condition and continuation in priming from Chinese to English (*β =* -13.84; *t* = -2.34) and in priming from English to Chinese (*β =* -7.92; *t* = -2.07): RTs were shorter for the SC continuation in the clustered than the anti-clustered condition, while no such effect was found for the DO continuation. This finding aligned with the prediction of the expectation adaptation account: participants exposed to clustered SCs in the training phase more strongly expected repetition of SCs in the test phase than those exposed to the same number of SCs spaced in time, whereas participants showed no differing expectation for repetition of DOs because this structure occurred only in the training phase and thus served as a baseline.

### Effects of L2 proficiency

To further explore the modulation of L2 proficiency on the two-way interaction between training condition and continuation, we added OQPT score as a continuous predictor to the fitted models. We predicted that the observed adaptation effect (i.e., faster processing for SCs given clustered environments) should be more robust with the increase of L2 proficiency, since more balanced bilinguals might possess stronger ability to adapt their expectation across languages. Our prediction was borne out: the processing advantage for SC continuation in the clustered training condition was more evident as L2 proficiency increased in both priming directions (Chinese-to-English: *β* = -11.10; *t* = -2.15; English-to-Chinese: *β* = -7.96; *t* = -2.09).

## Discussion

The aim of this study is to test the generalizability of the expectation adaptation mechanism in cross-linguistic syntactic priming and to investigate the influence of L2 proficiency on this mechanism. To this end, we employed a bilingual version of the self-paced paradigm of Myslín and Levy [12] which asked two groups of Chinese L2 learners of English to read the same number but different clustering properties of SCs in a training phase, and then required all of them to read repetitions of SCs in a test phase. Below we discuss the obtained results.

### Cross-linguistic expectation adaptation

Our results showed that participants with clustered training experience were more facilitated to process repetition of SCs in the subsequent test compared to those with anti-clustered training experience, despite repeated structures being presented in two different languages such as Chinese and English. This difference in cross-linguistic priming strength, similar to the findings of Myslín and Levy [12] and Han [13] in within-language priming, can be ascribed to cross-linguistic expectation adaptation: bilinguals are sensitive to environmental clustering properties of syntactic structures that are unspecific for languages, such that they can raise expectation for the repeated occurrence of a structure in environments where it clusters and lower such expectation in environments where it does not cluster. Accordingly, the adaptation of expectation for syntactic repetition leads to the variability in priming strength, which extends the expectation adaptation account to cross-linguistic syntactic priming.

A further question is why syntactic expectation can be adapted across languages. This question may find both language-specific and domain-general answers. On the one hand, it is possible that bilingual shared syntactic representations, in addition to the superficial lexical and syntactic properties (e.g., lemma, structural nodes, thematic roles, etc) [62,63], may also include more abstract and higher-order expectation for syntactic distributions. This possibility is well supported by the influence of syntactic frequency (e.g., the cumulative effect) [54–56] and clustering properties (our finding) on cross-linguistic priming strength. Leveraging the knowledge of such expectation is particularly beneficial to improve the efficiency and accuracy of sentence processing by facilitating resources allocation [35], reducing expected prediction errors [38], and negotiating considerable environmental variability [40]. On the other hand, the similar expectation adaptation has been widely observed in other linguistic levels except for syntax (e.g., phonetics [64], phonology [65], lexical associations [66], and semantic or pragmatic inferences [67], etc), and even in other cognitive domains beyond language (e.g., music [68], vision [69], motor control [70], and time perception [71], etc). The ubiquity of this behavior suggests that it may serve as a fundamental cognitive ability of the human brain, that is, to form prior expectations based on previous experience or knowledge and then update these expectations in the face of new information, which represents the Bayesian approach of cognitive processing [72,73].

### The influence of L2 proficiency

When incorporating L2 proficiency as an additional influencing factor, we found that the processing advantage for the clustered training group on SCs was larger with the increase of L2 proficiency in both priming from English to Chinese and the other way around. This finding indicates that more proficient L2 learners possess stronger ability to adapt expectation for syntactic repetition across languages, which enriches the expectation adaptation account in cross-linguistic syntactic priming by integrating the role of L2 proficiency.

This positive correlation between cross-linguistic expectation adaptation and L2 proficiency can be explained by drawing on the developmental theory of bilingual shared syntactic representations [19]. To be specific, in an early stage of L2 learning, bilinguals have a separate representation for L1 and L2 because they are not proficient enough in their L2. Then, as L2 learning progresses, the L2 representation gradually starts sharing with the existing L1 representation, mainly through the connections of structural nodes between languages. Finally, when bilinguals reach high proficiency in L2, they completely share representations of their two languages. This developmental theory is supported by research indicating that more balanced bilinguals show more comparable strength of within-language and between-language syntactic priming [74]. Although this theory is initially proposed based on the residual activation account that focuses on structural nodes, it can also be incorporated into the expectation adaptation account to explain syntactic expectations: expectation for a syntactic structure in L1 and L2 is initially constructed in separate, then get connected with each other as L2 proficiency grows, and eventually become fully shared between two languages as an endpoint of L2 learning trajectory. Hence, the greater degree of sharing expectations across languages naturally leads to the stronger capacity of adapting them.

Another plausible explanation for the proficiency pattern in our experiment can be derived from the increasing ability to engage in bilingual predictive processing. To illustrate, numerous studies have found that prediction or expectation in L2 syntactic processing is typically weaker than in L1 syntactic processing. For example, Spanish native speakers could use gender-marked determiners to expect the upcoming nouns, whereas L2 Spanish learners were less able or failed to do that [75,76]. This finding indicates that L2 learners have the reduced ability to generate expectations (RAGE) compared to L1 speakers [77]. The RAGE is argued to stem from the hypothesis that L2 learners are already overload by integrating the current syntactic information due to their processing being less automatic, and consequently possess limited cognitive resources available for expecting the upcoming syntactic information in L2 processing. Interestingly, several following studies have observed that more proficient L2 learners could generate expectations that were more native-like [78–80], which suggests that the RAGE can be improved with the increase of L2 proficiency. The reason for this improvement is that L2 processing become more automatic as L2 learning progresses, and thus more cognitive resources can be released to generate expectations. Following this logic, it is plausible that the ability to adapt expectations may also benefit from the increase of L2 proficiency because of more released cognitive resources, even in the cross-linguistic context.

### Towards an integrated mechanism of syntactic priming

As previously mentioned, our finding lends support to the expectation adaptation account by showing that participants experiencing different clustering properties of SCs exhibited varying strength of cross-linguistic priming. However, this differential priming cannot be explained by previous accounts of syntactic priming: given that all participants were exposed to the same total numbers of SCs, they should achieve the comparable residual activation of structural nodes or implicit learning of structural knowledge, thereby leading to no discernible difference in priming strength.

However, it is important to note that our aim is to merely verify the expectation adaptation account in cross-linguistic syntactic priming, but not to refute or preclude the potential contributions of residual activation and implicit learning account. In fact, these accounts are not necessarily in conflict, but rather in complement. Specifically, the expectation adaptation account refines the implicit learning account by showing that higher-order expectations for syntactic distributions are also learnable through experience. Some studies even manifest that the rational adaptation of expectation is an unified mechanism for both language processing and language learning [81–84], which reveals the potential of integrating the expectation adaptation and the implicit learning account. On the other hand, the expectation adaptation account supplements the residual activation account by offering an alternative processing mode. While the residual activation account reflects a bottom-up processing that starts with the specific structural nodes and gradually moves up to higher levels of syntactic representations, the expectation adaptation account represents a top-down processing that highlights comprehenders’ sensitivity to distributional patterns of syntactic structures in broader linguistic environments. These two processing modes are not mutually exclusive and thus may work in an interactive way in syntactic priming. However, future studies are required to provide more evidence for such interaction.

The complementary nature of these three accounts is also evident in other effects or characteristics related to syntactic priming. For example, considering the discovery that lexical properties also cluster in natural language [85], the expectation adaptation account predicts that the lexical boost effect (i.e., priming strength is enhanced when the target verb repeats the prime verb) may simply be an optimal response to lexical clustering in the environment. However, this prediction does not adequately explain why the lexical boost effect is often restricted to repetition of the verb, rather than for any content word in the sentence [86]. Conversely, this restriction can be more aptly explained by the residual activation account which posits that since structural nodes are connected to verbs, only verb repetition can activate this connection to enhance priming effects. In addition, the expectation adaptation account posits no predictions regarding the duration of syntactic priming, whereas the short-term priming is viewed as a result of transient residual activation and the long-term priming is interpreted as an outcome of persistent implicit learning.

Given the foregoing, it is reasonable to infer that syntactic priming may be driven by an integrated mechanism that incorporates the residual activation, implicit learning and expectation adaptation collectively. Recognizing and exploring this integrated mechanism holds significant implications for future research, potentially offering a more comprehensive and nuanced understanding of syntactic priming.

### Limitations and suggestions

To start with, this study only focuses on the short-term and lexically independent expectation adaptation. Future research may investigate whether temporary adaptation can lead to persistent retention and whether lexical overlaps can enhance the strength of expectation adaptation. Additionally, this study merely uses the self-paced reading paradigm with facilitated reading time as an indicator of syntactic priming. In fact, syntactic priming in comprehension is also indicated by anticipatory eye movements to an expected referent, P600 waves in the ERP (event-related potential), and changes in the BOLD (blood oxygenation level dependent) response during fMRI [11]. Future research can employ other paradigms to enrich the investigation. Moreover, this study explores the expectation adaptation without time limits. However, some studies have shown that for tasks with very rapid timescales, it may not be feasible to learn and deploy knowledge of environmental pattern in real time [87,88]. Future research may manipulate the response intervals to explore the expectation adaptation in tasks with different timescales.

## Conclusion

By employing a bilingual version of the self-paced reading paradigm of Myslín and Levy [12] with Chinese L2 learners of English, we demonstrated that bilinguals rationally adapt expectation for repetition of a structure based on its environmental clustering properties across languages, and that the ability to make such adaptation is enhanced as L2 proficiency increases. Overall, our findings enriches the mechanistic understanding of syntactic priming by extending the expectation adaptation account to the cross-linguistic context with the modulation of L2 proficiency. Additionally, our findings are also informative for bilingual shared syntactic representations by showing that abstract, higher-order distributional expectations for syntactic structures can be shared between languages, and for the expectation-based language comprehension by pinpointing the adaptive nature of cross-linguistic syntactic priming.

## Supporting information

S1 Data (CSV)
